# Respiratory Muscle Training in Patients Recovering Recent Open Cardiothoracic Surgery: A Randomized-Controlled Trial

**DOI:** 10.1155/2013/354276

**Published:** 2013-07-30

**Authors:** Ernesto Crisafulli, Elena Venturelli, Gherardo Siscaro, Fabio Florini, Alessandra Papetti, Daniela Lugli, Massimo Cerulli, Enrico Clini

**Affiliations:** ^1^University of Modena, DU of Medical and Surgical Sciences, Via del Pozzo 171, 41121 Modena, Italy; ^2^Villa Pineta Hospital, Lung Unit and Pulmonary Rehabilitation, Via Gaiato 127, 41020 Pavullo nel Frignano, Modena, Italy

## Abstract

*Objectives*. To evaluate the clinical efficacy and feasibility of an expiratory muscle training (EMT) device (Respilift) applied to patients recovering from recent open cardiothoracic surgery (CTS). *Design*. Prospective, double-blind, 14-day randomised-controlled trial. *Participants and Setting*. A total of 60 inpatients recovering from recent CTS and early admitted to a pulmonary rehabilitation program. *Interventions*. Chest physiotherapy plus EMT with a resistive load of 30 cm H_2_O for active group and chest physiotherapy plus EMT with a sham load for control group. *Measures*. Changes in maximal expiratory pressure (MEP) were considered as primary outcome, while maximal inspiratory pressures (MIP), dynamic and static lung volumes, oxygenation, perceived symptoms of dyspnoea, thoracic pain, and well being (evaluated by visual analogic scale—VAS) and general health status were considered secondary outcomes. *Results*. All outcomes recorded showed significant improvements in both groups; however, the change of MEP (+34.2 mmHg, *P* < 0.001 and +26.1%, *P* < 0.001 for absolute and % of predicted, resp.) was significantly higher in active group. Also VAS dyspnoea improved faster and more significantly (*P* < 0.05) at day 12, and 14 in active group when compared with control. The drop-out rate was 6%, without differences between groups. *Conclusions*. In patients recovering from recent CTS, specific EMT by Respilift is feasible and effective. This trial is registered with ClinicalTrials.gov NCT01510275.

## 1. Introduction

In the postoperative period patients undergoing cardiothoracic surgery (CTS) have an increasing risk to develop pulmonary complications such as atelectasis, pneumonia, or pleural effusion, potentially leading to increasing length of stay and rate of hospital mortality [1, 2]; in these patients symptoms (dyspnoea and pain) are usually perceived as disabling, both linked to the reduction of lung ventilation following the chest opening.

 Pulmonary rehabilitation (PR) is a nonpharmacological intervention aimed at improving exercise tolerance, symptoms, and general health status in patients with chronic respiratory diseases [3, 4]. In subjects recovering recent thoracopulmonary surgery for lung resection, pleural decortication [5, 6], or coronary artery bypass graft (CABG) [7, 8], the application of a rehabilitation course including specifically pulmonary reexpansion and/or respiratory muscle training represents a potentially useful intervention, especially if applied in a period less than one week after the surgery (e.g., when patients were directly transferred from surgical unit) [5, 9, 10]. 

In mild to very severe patients suffering from chronic obstructive pulmonary disease (COPD), the application of two recent easy-to-use respiratory devices (Respivol and Respilift) has been shown to enhance the pulmonary volumes, the respiratory muscle performance (maximal inspiratory pressure (MIP) and maximal expiratory pressure (MEP)), and the perceived dyspnoea when used alone [11] or even in combination [12].

Aim of our study was to evaluate the clinical efficacy and feasibility of specific expiratory muscle training (EMT) by the Respilift device applied to patients recovering from recent open CTS. 

## 2. Methods

### 2.1. Study Design and Patients

**Figure 1:**
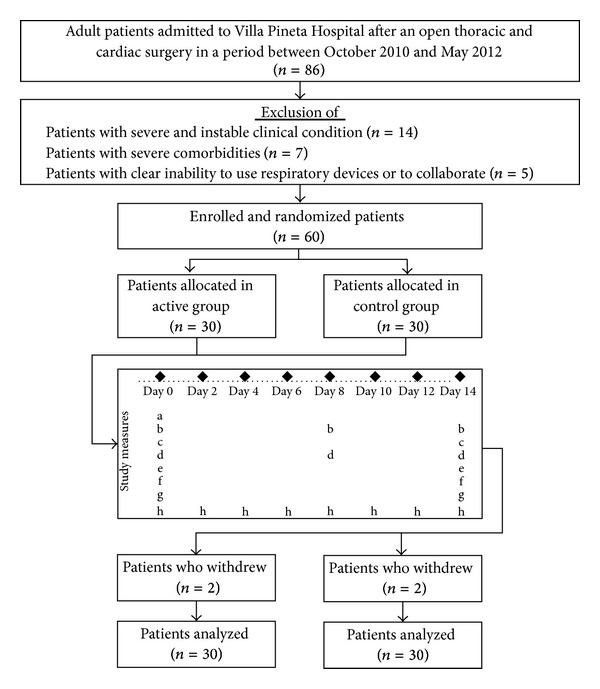
Study flow diagram. Study measures collected by demographic data (a), pletismography (b), arterial gas analysis (c), respiratory muscle performance (d), 6 min walking test (e), chronic MRC dyspnoea scale (f), health status-SF 36 (g), and patient-related symptoms (dyspnoea, thoracic pain, and well being) (h).

This was a prospective, double-blind, parallel-assignment, AND randomized-controlled study carried out at an Italian rehabilitation centre (Villa Pineta Hospital, Pavullo nel Frignano, Modena, Italy) for a period of 14 consecutive days. The hospital board approved the trial, which was conducted in accordance with the declaration of Helsinki and good clinical practice rules and recorded on ClinicalTrial.gov website with identification code NCT01510275. Selected patients gave their written informed consent to participate in the study.  Figure 1 shows the study flow diagram, according to CONSORT statement for nonpharmacologic treatment [13]. 

 In a period between October 2010 and May 2012 we enrolled and consecutively selected 60 adult patients admitted to an inpatient PR course one to three weeks after open CTS. At the enrolment, patients with severe or unstable clinical condition (e.g., respiratory distress) or comorbidities requiring strict medical monitoring (e.g., congestive heart failure) were excluded from the study. Patients showing inability to use the respiratory device or to collaborate with the attending physiotherapist were excluded also. Patients with new occurring clinical signs and worsening condition after enrolment were considered as dropouts. 

### 2.2. Interventions

**Figure 2:**
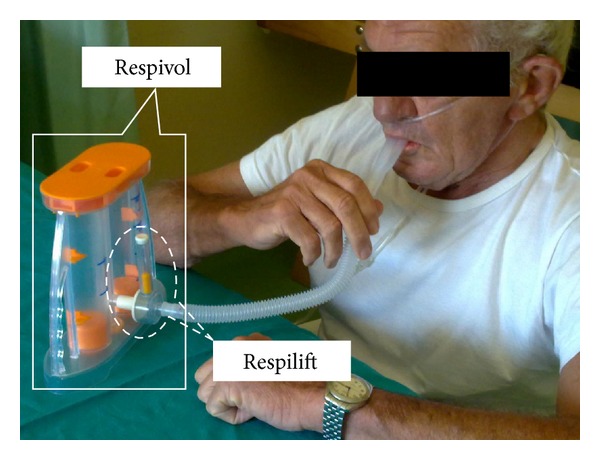
Representative patient using the combined devices Respivol and Respilift.

 By a monoblock electronically generated list at the enrolment patients were randomly allocated to the active or control group. Patients allocated in active group used Respilift, a device developing EMT with a resistive load of 30 cm H_2_O integrated into and in conjunction with Respivol, a volume-targeted incentive spirometer (see Figure 2); patients in control group used the same devices but with a sham load (no resistance) for Respilift. Each training session consisted of 15 minutes and was performed twice a day; a respiratory physiotherapist (deputed to the study), unaware of the study aim and group allocation, assisted all enrolled patients. With the intent to familiarizing with Respilift and Respivol eligible patients were instructed to use devices for a 2-hour training period. 

In the morning of each day of the study, by a pressure manometer, Respilift was previously tested in order to define an accurate resistive load. Inspiratory volume as well as the inspiratory flow were set on two calibrated scales and visually displayed by plates which are lifted up and kept suspended by a sustained, slow, and deep inspiration. Expiratory resistive load of 30 cm H_2_O has been applied by a disposable blinded plastic column with one-way valve and containing a floating mobile indicator which defined the target load for the patients. A mouthpiece tube endowed with a filter connected this resistor to one side of the volume chamber. 

 Chest physiotherapy program (similarly for active and control groups) was performed by manually assisted lung expansion (Exercise à Debit Inspiratoire Controlè (EDIC) and Expiration Lente Totale Glotte Ouverte en Infralatéral (ELTGOL)) as recommended [3, 6] for 20 consecutive minutes twice a day; a short preliminary description of the procedures was given by the attending physiotherapist, previously instructed to homogenise the type and duration of all the activities.

### 2.3. Measurements and Outcomes

 At enrolment (day-0) demographics and general characteristics were collected. Outcome measures were recorded at day 8 and at the end of study (day 14) while data on individuals' perceived symptoms (dyspnoea, thoracic pain, and well being) were recorded every 2 days from the enrolment (day 0, day 2, day 4, day 6, day 8, day 10, day 12, day 14).

#### 2.3.1. Primary Outcome

 Maximal expiratory pressures (MEP) were performed by means of a specific module (Masterscope; Jaeger; Hoechberg, Germany) recording maximal pressures against an occlusive mouth resistance at both total lung capacity and functional residual volume; values were recorded as absolute values (mmHg) and as percentage (%) of predicted according to the reference equations [14], and the best of three measurements was considered for the study.

#### 2.3.2. Secondary Outcomes

The same module and modalities of MEP were used to measure maximal inspiratory pressure (MIP) but maximal pressures against an occlusive mouth resistance at functional residual volume; values were recorded as absolute values (mmHg) and as percentage (%) of predicted.

 Forced and static lung capacities and volumes (FEV_1_: forced expiratory volume in the first second, FVC: forced vital capacity, VC: vital capacity, IC: inspiratory capacity, TLC: total lung capacity, and RV: residual volume) were taken by pletismography (Pletismography Platinum DX, MedGraphics, Saint Paul, MN, USA), expressed both in absolute values (litres) and as percentage of predicted [15].

 With the patient breathing in a room air, a radial arterial gas analysis was performed by an automated analyzer (Model 850; Chiron Diagnostics; Medfield, MA, USA): measurements of arterial carbon dioxide pressure (PaCO_2_), arterial oxygen pressure (PaO_2_), and the ratio of partial arterial oxygen pressure to the fraction of inspired oxygen (PaO_2_/FiO_2_) were collected.

According to the American Thoracic Society (ATS) statement [16] a 6 min walking test (6 MWT) was performed to evaluate exercise capacity; moreover, chronic dyspnoea (by medical research council (MRC) score) [17] and individual's health-related status (by SF36: short form 36 health survey questionnaire) [18] were detected as pre-to-post (on day 0 and day 14) measures of the rehabilitation course.

 A time trend (change) in visual analogic scale (VAS) [19] was used to assess perceived sensation of dyspnoea, thoracic pain, and well being. 

### 2.4. Statistical Analysis

Analysis of study variables was performed using a statistical software package (SPSS 17 for Windows).

 Based on our preliminary data in similar patient [5], a minimum sample size of 27 patients per arm was required in order to obtain a statistical power of 90%, to be able to detect a difference between groups after intervention of 10 cm H_2_O in the primary outcome.

All data was evaluated in terms of the intention-to-treat approach (ITT); last observation carried forward (LOCF) was used as a method of ITT and data are presented accordingly [20].

Results were expressed as mean ± standard deviation (SD) or 95% confidence interval (CI) for continuous variables and frequency (percentage) for categorical variables. Test for normality of data distribution was performed (Kolmogorov-Smirnov test) *a priori.* Differences in the continuous variables were then analyzed using an independent two-tailed *t*-test for unpaired variables; otherwise, the nonparametric Mann-Whitney *U* test was used when appropriate. Categorical variables were studied using the *χ* ^2^ test or Fisher's exact test when necessary.

For all analyses, an *α*-error <5% was considered to be statistically significant.

## 3. Results

 Sixty patients in active (*n* = 30) and control (*n* = 30) groups represented the study cohort. All of them were recently admitted to our centre after open cardiac (50 patients, 83%) or thoracic (10 patients, 17%) surgery. No differences in main anthropometric, clinical, and functional characteristics were reported between groups (Tables 1 and 2). A coexisting diagnosis of COPD was present in 10%, and in 5 of these patients (8%) long-term oxygen therapy (LTOT) treatment was required. Overall, the drop-out rate was 6%, without any group differences. 

**Figure 3:**
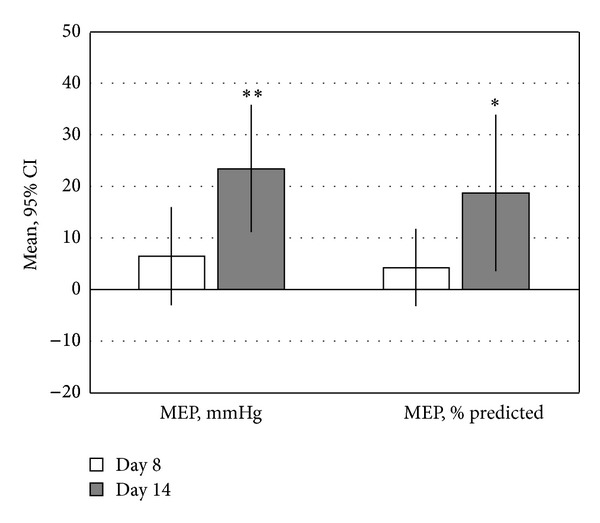
Treatment difference (active versus control group) in the primary study outcome evaluated at day 8 and day 14. MEP: maximal expiratory pressure. *P < 0.05; **P < 0.001.

Table 2 shows pulmonary, exercise capacity, and quality of life during the training period. After 14 days improvements in dynamic lung volumes (FEV_1_, FVC, and VC), respiratory muscle strength (MIP and MEP), gas exchange (PaO_2_/FiO_2_), walking capacity at 6 MWT, and chronic dyspnoea scale (MRC) were recorded in both groups; moreover, the improvement of maximal expiratory performance (MEP), measured as group difference both in absolute and % change between enrolment and day 8 and day 14, was significantly higher in active group (see Table 2 and Figure 3). 

**Figure 4:**
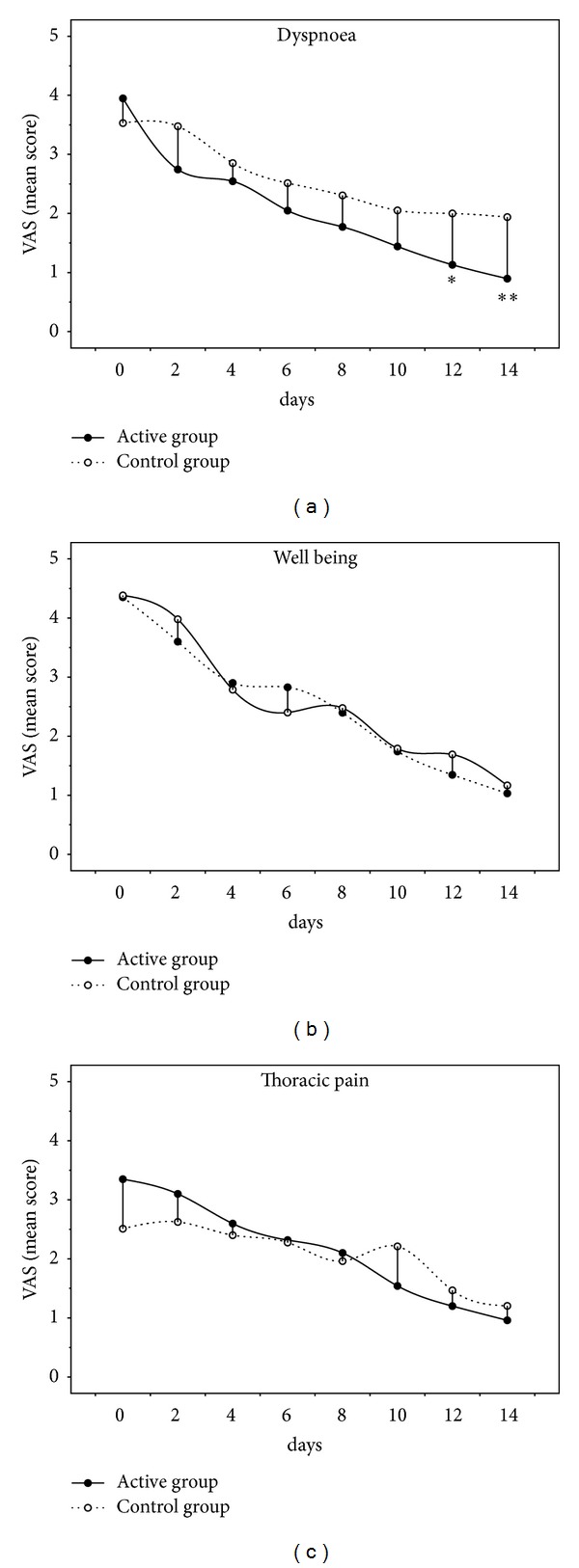
Trend of symptoms reported by VAS score. VAS: visual analogic scale. ^∗,∗∗^ *P* < 0.05 and *P* < 0.001 between groups at the same time, respectively.

 The daily trend of VAS dyspnoea, well-being and thoracic pain, showed a similar and progressive improvement over time in both groups: however, VAS dyspnoea improved faster and more significantly at day 12 and day 14 of treatment in the active when compared with control group (see Figure 4). Moreover, the EMT did not worsen the thoracic pain, nor did it alter its recovery course. 

## 4. Discussion

Respilift, a respiratory device specifically aimed at EMT and added to chest physiotherapy, has proved to be effective over a 14-day period in patients recovering from CTS when compared with patients performing a EMT with a sham load. The faster reduction of perceived dyspnoea together with the absence of any negative interference on the recovery of thoracic pain also proved feasibility and safety of the device. 

 Patients undergoing CTS may develop significant pulmonary derangements and complications related to the reduction of lung expansion and compliance; clinically, in these patients dyspnoea and pain are the dominant symptoms, especially during minimal efforts. Moreover, these pulmonary pathophysiological alterations are associated with the risk of prolonging the hospital stay following surgery [1, 2, 21]. Even if there are no consolidated scientific evidences in this population, it is not clear whether surgical patients are suitable candidates for a postoperative rehabilitation course including chest physiotherapy [22–25]. Some clinical trials showed this treatment as effective in improving pulmonary expansion and respiratory muscle performance, furthermore achieving a subsequent increase of physical capacities, gas exchange, and quality of life perceived in the individual patient [5–8]. 

 To our knowledge there is only one experience [11, 12] demonstrating that a 1-year home-based program including combined lung expansion and expiratory muscle training was associated with progressive improvements of MIP and MEP and dyspnoea perception, in moderate COPD patients. Our prospective study is the first showing that combination of a lung expansion device (Respivol) and EMT (Respilift) over 14 days is functionally and clinically associated with more benefits than a sham training of Respilift; for both groups chest physiotherapy with lung expansion was the only technique used in the clinical practice in the population of individuals recovering from cardiac or thoracic open surgery.

 Thoracic expansion *per se* is clearly associated with improvement of both pulmonary volumes and oxygenation [22], general physical functions, and symptoms [23], thus confirming the clinical usefulness of this chest physiotherapy technique following thoracic or cardiac surgery, as preliminary suggested by retrospective data in our centre [5]. In addition, the selective use of a medium-term resistive EMT course by the Respilift was associated with additional and specific improvement of performance only in those muscle groups which were trained (see Table 2 and Figure 3).

 A significant point in favour of the use of EMT could be considered the drop of individual's symptoms recorded over the study period, which was faster (from day 12 on) for dyspnoea when the combination of techniques was used in comparison with the use of volume-targeted lung expansion alone (see in Figure 4). This result seems of true clinical usefulness to those patients, since dyspnoea and thoracic pain are the most relevant complaints after surgery [5, 23]. Interestingly enough, the reduction of the VAS score in thoracic pain during the active EMT period paralleled that recorded during sham resistive training and chest physiotherapy. Indeed, one could have potentially expected that training the expiratory muscle against a substantial resistive load (30 cm H_2_O) might have been associated with increasing pain or higher dropout rate, which was not the case in our study. This result stands for a safety use of this training device in this population after surgery with the aim to accelerate the fact that the individual's functional recovery and the reduction of symptoms which may limit lung function, cough reflex and consequently favour the late pulmonary complication in the predisposed individuals [22]. Notwithstanding, we missed any specific evaluation of cough performance (e.g., peak cough expiratory flow) which might have reinforced this concept. 

 Despite the interesting and positive results in our trial, these should be viewed with caution by readers due to several limitations that need to be addressed. First, we did not know the level of respiratory muscle performance before admission to surgery; despite the post-training level of both MIP and MEP fell in the range of *quasi*-normality, it is likely that the starting level of respiratory muscle performance may be a factor conditioning the change after training. As a matter of fact around 10% of the patients in study had associated COPD; thus they might have more benefit following specific training of their inspiratory muscles [26]. Moreover, the percentage of MIP load were patients working at was not measured before, and we cannot exclude that changes after training would have been influenced by different level of inspiratory muscle load. Nonetheless, our study specifically aimed at training expiratory muscles that is the reason why changes in intervention and control group significantly differed at the end of the trial for MEP only and not for MIP. Since the population was composed by patients recovering cardiac surgery with only a minimal part of them being COPD, it would be very difficult to speculate over effectiveness on MIP, even in comparison with previous studies.

Second, the present findings were obtained once the patients were stabilized and transferred from surgical units to rehabilitation in a period between one to three weeks; therefore, we cannot extrapolate the results (both in terms of efficacy and feasibility) in a clinical phase closer to the surgical intervention. Nonetheless, we cannot exclude a spontaneous (although partial) recovery of patient's functions in the phase between surgery and actual physiotherapy. Finally and additionally, since this study lacks a long follow-up period, we cannot exclude that obtained results would have been maintained over weeks or months after the effective training. A specific study would be able to answer this question.

## 5. Conclusions

 In conclusion this preliminary and controlled study has shown that an easy-to-use respiratory device to train expiratory muscle has additional effects when compared with a sham training in the population of patients recovering the open thoracic or cardiac surgery. 

## Figures and Tables

**Table 1:** General characteristics of patients enrolled.

Variables	Active group (*n* = 30)	Control group (*n* = 30)	*P*
Age, years	66.8 ± 7.3	67.0 ± 9.8	0.941
Sex, M/F	24/6	20/10	0.243
BMI, kg·m^2^	25.4 ± 3.3	25.7 ± 4.1	0.727
COPD	2 (6)	4 (13)	0.389
LTOT	2 (6)	3 (10)	0.640
FiO_2_^†^, %	28.0 ± 5.6	32.0 ± 4.0	0.413
Drop-out	2 (6)	2 (6)	1
Cardiothoracic surgical intervention			0.622
CABG	10 (33)	7 (23)	
Aortic valve replacement	8 (26)	11 (36)	
Mitral valve repair	4 (13)	5 (16)	
Mitral valve replacement	2 (6)	3 (10)	
Pulmonary lobectomy	4 (13)	3 (10)	
Pulmonary resection	2 (6)	0 (0)	
Pneumonectomy	0 (0)	1 (3)	

Variables described as mean ± SD or as frequency (%).

^†^Defined in LTOT patients only.

BMI: body mass index; COPD: chronic obstructive pulmonary disease; LTOT: long-term oxygen therapy; FiO_2_: inspiratory fraction of oxygen; CABG: coronary artery bypass graft.

**Table 2:** Study outcomes evaluated as change from the baseline.

	Baseline		Change from baseline at day 8		Change from baseline at day 14	
Variables	Active group (*n* = 30)	Control group (*n* = 30)	Active group (*n* = 30)	Control group (*n* = 30)	Active group (*n* = 30)	Control group (*n* = 30)
FEV_1_, litres	1.9 ± 0.5	1.6 ± 0.6	0.0 ± 0.3 (0)	0.0 ± 0.2 (0)	0.1 ± 0.3 (5)*	0.1 ± 0.2 (6)*
% predicted	69.8 ± 18.4	64.7 ± 15.0	3.6 ± 10.2 (5)	2.6 ± 8.5 (4)	6.5 ± 10.1 (9)*	6.0 ± 10.6 (9)*
FVC, litres	2.4 ± 0.7	2.1 ± 0.9	0.1 ± 0.5 (4)	0.0 ± 0.3 (0)	0.2 ± 0.6 (8)*	0.2 ± 0.4 (10)*
% predicted	69.7 ± 15.0	66.3 ± 15.3	4.9 ± 11.2 (7)*	4.5 ± 11.8 (7)	8.8 ± 13.0 (13)**	8.3 ± 12.6 (13)*
VC, litres	2.5 ± 0.5	2.3 ± 0.7	0.1 ± 0.3 (4)*	0.0 ± 0.4 (0)	0.2 ± 0.2 (8)**	0.3 ± 0.4 (13)*
% predicted	68.3 ± 11.7	62.6 ± 13.6	3.7 ± 7.1 (5)*	3.6 ± 11.4 (6)	4.3 ± 5.1 (6)**	6.6 ± 12.3 (11)*
IC, litres	2.8 ± 3.8	1.8 ± 0.7	−0.6 ± 3.8 (−21)	0.1 ± 0.6 (6)	−0.5 ± 3.8 (−18)	0.2 ± 0.7 (11)
% predicted	74.4 ± 16.0	70.1 ± 14.7	3.4 ± 12.3 (5)	3.1 ± 13.2 (4)	5.1 ± 11.7 (7)*	6.5 ± 17.0 (9)
TLC, litres	5.4 ± 1.0	4.9 ± 1.2	0.2 ± 0.6 (4)	0.2 ± 0.9 (4)	0.1 ± 0.8 (2)	0.4 ± 1.0 (8)
% predicted	86.6 ± 13.1	84.6 ± 15.5	3.2 ± 10.3 (4)	3.5 ± 10.7 (4)	6.9 ± 13.5 (8)*	4.1 ± 15.9 (5)
RV, litres	2.7 ± 0.9	2.2 ± 1.1	−0.1 ± 1.0 (−4)	0.1 ± 0.8 (5)	−0.0 ± 0.9 (0)	0.3 ± 1.0 (14)
% predicted	113.4 ± 37.2	97.5 ± 44.0	−7.1 ± 39.5 (−6)	6.3 ± 37.0 (6)	1.1 ± 29.8 (1)	15.7 ± 35.5 (16)*
MIP, mmHg	59.9 ± 28.9	53.4 ± 21.3	8.1 ± 13.9 (14)*	7.5 ± 15.3 (14)*	13.3 ± 23.1 (22)*	13.4 ± 18.6 (25)*
% predicted	60.5 ± 28.4	56.0 ± 20.5	9.5 ± 17.6 (16)*	5.6 ± 16.3 (10)	13.5 ± 22.5 (22)*	13.0 ± 16.5 (23)*
MEP, mmHg	83.5 ± 23.7	76.8 ± 30.8	15.1 ± 16.1 (18)**	8.7 ± 17.5 (11)*	34.2 ± 24.9 (41)**	10.8 ± 18.3 (14)*
% predicted	46.0 ± 14.6	43.5 ± 16.5	7.6 ± 10.7 (17)**	3.3 ± 15.5 (8)	26.1 ± 32.4 (57)**	7.4 ± 19.9 (17)
PaO_2_/FiO_2_, %	343.3 ± 47.8	337.6 ± 51.5	—	—	22.9 ± 34.6 (7)*	32.4 ± 52.6 (10)*
PaCO_2_, mmHg	34.2 ± 4.8	35.5 ± 5.2	—	—	0.8 ± 3.8 (2)	−0.7 ± 4.4 (−2)
Distance walked at 6 MWT, meters	308.9 ± 86.7	295.3 ± 108.4	—	—	125.6 ± 76.1 (41)**	108.0 ± 74.7 (37)**
MRC, score	2.1 ± 0.5	2.0 ± 0.5	—	—	−1.0 ± 0.8 (−48)**	−0.8 ± 0.3 (−40)**
SF36FI, score	32.7 ± 6.6	33.5 ± 8.1	—	—	3.2 ± 6.4 (10)*	4.5 ± 8.9 (13)*
SF36ME, score	41.0 ± 11.0	41.5 ± 10.7	—	—	3.9 ± 5.8 (10)*	3.2 ± 10.5 (8)

Variables described as mean ± SD. In parenthesis percentage of change from baseline.

No significant differences between active and control groups are reported in all baseline variables considered.

FEV_1_: forced expiratory volume in the first second; FVC: forced vital capacity; VC: vital capacity; IC: inspiratory capacity; TLC: total lung capacity; RV: residual volume; MIP: maximal inspiratory pressure; MEP: maximal expiratory pressure; PaO_2_/FiO_2_: arterial oxygen pressure on inspiratory oxygen fraction, ratio; PaCO_2_: arterial carbon dioxide pressure; 6 MWT: 6 min walking test; MRC: medical research council; SF36 PC and MC: short form 36 health survey questionnaire, physical and mental component, respectively.

*P < 0.05; **P < 0.001.
